# A simple, rapid typing method for *Streptococcus agalactiae* based on ribosomal subunit proteins by MALDI-TOF MS

**DOI:** 10.1038/s41598-020-65707-5

**Published:** 2020-05-29

**Authors:** Julian Rothen, Dulmini Nanayakkara Sapugahawatte, Carmen Li, Norman Lo, Guido Vogel, Frédéric Foucault, Valentin Pflüger, Joёl F. Pothier, Jochen Blom, Claudia Daubenberger, Margaret Ip

**Affiliations:** 1 Department of Medical Parasitology and Infection Biology, Swiss Tropical and Public Health Institute, Basel, Switzerland; 2 University of Basel, Basel, Switzerland; 3 Department of Microbiology, The Chinese University of Hong Kong, Shatin, Hong Kong; 4 Mabritec AG, Riehen, Switzerland; 5 Research Group for Environmental Genomics and Systems Biology, Institute of Natural Resource Sciences, Zurich University of Applied Sciences (ZHAW), Wädenswil, Switzerland; 6 Bioinformatics and Systems Biology, Justus-Liebig-Universität Gießen, Giessen, Germany

**Keywords:** Microbiology, Medical research

## Abstract

*Streptococcus agalactiae* (Group B *Streptococcus*, GBS), is a frequent human colonizer and a leading cause of neonatal meningitis as well as an emerging pathogen in non-pregnant adults. GBS possesses a broad animal host spectrum, and recent studies proved atypical GBS genotypes can cause human invasive diseases through animal sources as food-borne zoonotic infections. We applied a MALDI-TOF MS typing method, based on molecular weight variations of predefined 28 ribosomal subunit proteins (rsp) to classify GBS strains of varying serotypes into major phylogenetic lineages. A total of 249 GBS isolates of representative and varying capsular serotypes from patients and animal food sources (fish and pig) collected during 2016–2018 in Hong Kong were analysed. Over 84% (143/171) noninvasive carriage GBS strains from patients were readily typed into 5 globally dominant rsp-profiles. Among GBS strains from food animals, over 90% (57/63) of fish and 13% (2/15) of pig GBS matched with existing rsp-profiles, while the remainder were classified into two novel rsp-profiles and we failed to assign a fish strain into any cluster. MALDI-TOF MS allowed for high-throughput screening and simultaneous detection of novel, so far not well described GBS genotypes. The method shown here is rapid, simple, readily transferable and adapted for use in a diagnostic microbiology laboratory with potential for the surveillance of emerging GBS genotypes with zoonotic potential.

## Introduction

*Streptococcus agalactiae* (Group B *Streptococcus*, GBS), is a frequent colonizer of the human gastrointestinal and genitourinary tracts^1^. GBS possesses a broad animal host spectrum including cattle^2^, pigs^3^, camel^4^ and various freshwater fish species^5,6^. Besides being a leading cause of neonatal meningitis and sepsis^7^, GBS is an emerging infectious disease in non-pregnant adults, and in the elderly^8,9^. GBS disease in non-pregnant adults prerequisites the switch of GBS from a harmless commensal to an invasive pathogen, a mechanism that remains poorly understood^10^. However, there is increasing evidence that GBS disease can also occur through nosocomial and food-borne infection^11^.

The GBS clone belonging to Sequence type ST283 exemplifies the threat of zoonotic infection in adults. During 1993–2012, this clone accounted for a significant number of invasive disease cases in non-pregnant adults in Hong Kong^8,12^. ST283 was previously described as a disease-causing strain in farmed freshwater fish^6^, and subsequent proof was confirmed in 2015 when ST283 was linked to an outbreak of adult GBS infections in Singapore, unequivocally linking to the consumption of raw fish^11^. Genomic analysis of human and fish ST283 strains later confirmed freshwater fish as reservoir of ST283, declaring this zoonotic clone a major infectious disease threat^13^. Large-scale epidemiological studies will be essential to gain insight into GBS transmission dynamics, in particular regarding the significance of animal reservoirs for emerging hyper-virulent GBS clones.

Matrix-assisted laser desorption/ionization time-of-flight mass spectrometry (MALDI-TOF MS) has developed into a widely used method for high-throughput microbial species identification in routine diagnostics^14,15^. Classifying microbial species based on their highly specific whole-cell peptide fingerprint, MALDI-TOF MS can be used to accurately discriminate between thousands of bacteria, including GBS^16,17^. The genetic diversity of GBS was found to be concurrent with variations in the protein patterns measured by MALDI-TOF MS^18^, which can be exploited for sub-species level discrimination of GBS strains^19^. We recently expanded on these findings and showed that the highly conserved ribosomal subunit proteins (rsp) serve as ideal biomarkers for strain level typing of GBS^20^. Specifically, indexing the mass variations of pre-defined 28 rsp using MALDI-TOF MS allows for classification of GBS isolates into the major phylogenetic lineages, detection of hyper virulent CC17 strains and identification of obligate animal associated lineages^20^.

We further applied the rsp-based MALDI-TOF MS approach in this study to (i) analyze the major GBS serotypes circulating among hospital patients during 2016–2018 and (ii) to identify the major GBS genotypes found in fish and pig meat samples collected from Hong Kong wet markets. This analysis will provide insights on (a) whether GBS genotypes circulating in humans and food animals differ within the city and (b) whether MALDI-TOF MS can be used as a high-throughput and cost-efficient screening tool for monitoring of emerging, potential zoonotic GBS clones. Here we have analysed a collection of 249 GBS isolates using a simple sample preparation method. Our results support the idea that our GBS typing approach based on measurement of 28 rsp which can be transferred between different laboratories to incorporate into routine diagnostic microbiological laboratories.

## Materials and methods

### Bacteria selection and serotypes

A total of 249 GBS samples isolates collected from human (N = 171), fish (N = 63) and pigs (N = 15) were analysed in this study. All animal GBS samples were collected during 2016–2018 from wet markets across Hong Kong in our food sample screening project and 171 non-duplicate human GBS isolates were cultivated and stored during 1st January to 31^st^ May 2018 from patients admitted to Prince of Wales Hospital, Shatin, Hong Kong. The informed written consents were obtained from the patients and any subject below 18 years, the informed written consents were obtained from the parents or/and guardian before collecting the samples. Ethics approval of the human GBS strains were obtained from the local ethics committee (The Joint Chinese University of Hong Kong – New Territory East Cluster Clinical research Ethics committee - CUHK-NTEC CREC-Ref No. 2019.013) and all clinical samples were tested according to the guidelines of ethical approval.

GBS strains were grown overnight at 37 °C with 5% CO_2_ on blood agar (OXOID, United Kingdom) and species identity was confirmed by the microflex LT MALDI-TOF MS system against the commercial reference spectra database (Bruker Daltonics, Bremen, Germany) prior to performing DNA extraction by simple boiling method. Capsular polysaccharide serotyping was done by multiplex PCR as previously described^21^ and any isolates that failed to be assigned to a serotype by visual assessment of the PCR bands were grouped as non-typeable (NT).

## MALDI-TOF MS Analyses

### Sample pre-processing

Bacterial colonies grown overnight on 5% blood agar were suspended in TMA buffer (10 mM Tris–HCl (pH 7.8), 30 mM NH4Cl, 10 mM MgCl2, and 6 mM 2-mercaptoethanol). Approx. 3×1 µl loopful of bacterial colonies were suspended in 500 µl TMA buffer in an 1.5 ml Eppendorf and washed thrice in TMA buffer. The bacterial cells were then disrupted to release the intracellular proteins by addition of 0.1 mm glass beads (Thermo Fisher Scientific, USA) to a micro tube, and placed on a FastPrep FP120 bead beater (Disruptor Genie, USA) and agitated for multiple 20 s interval at maximum speed, with cooling intervals of 1 min on ice. Protein fragments smaller than 3,000 Da were removed by filtering of the bacterial extract with Ultra centrifugal devices (Amicon, 0.5 ml, 3k Da) and the concentrated protein samples were diluted tenfold in ddH_2_O as previously described^20^. The final protein solution was spotted in quadruplicates on the MALDI-TOF MS target plate and overlaid with 1 µl sinapinic acid matrix solution (10 mg sinapinic acid in 60% acetonitrile, 40% ddH_2_O and 1% TFA).

### Microflex instrument setup

The MS measurements were carried out on a microflex LT MALDI-TOF MS system (Bruker Daltonics, Bremen, Germany) with detection in the linear mode, allowing the interrogation of high molecular weight samples. The instrument parameter settings were adjusted to be on par with the use of sinapic acid matrix. The linear detector voltage was set to 1,943 V, with a laser frequency of 66.7 Hz, initial laser power of 70%, maximal laser power of 90% and laser attenuation offset of 60%. For each spectrum, 1,000 laser shots in 100 shot steps were acquired (FlexControl Software 2.0, Bruker Daltonics) in a random walk movement, thereby ensuring an even measurement covering the entire area of the sample spot. Each stainless steel target plate was externally calibrated using the reference spectra of *Escherichia coli* strain DH5α.

### Spectra post-processing and internal calibration

Post-processing of the raw mass spectra (fid files) was carried out using a custom R script, building on the R package MALDIquant^22^. Briefly, peak intensities were square root transformed and smoothed using the “SavitzkyGolay” method. Baseline removal was done using the “SNIP” baseline estimation method. Peak detection was carried out using the “MAD” noise estimation method and a signal-to-noise ratio of 2. Internal calibration with 800 ppm was carried out using 10 GBS rsp masses (3 mass alleles of L6, 2 mass alleles of L36 and S12, 1 mass allele of L14, L29 and S15) that altogether display molecular weights distributed over a wide mass range (4,425 to 19,293 Da). An ascii file containing the recalibrated protein mass values and corresponding intensities was automatically generated for every mass spectrum.

### Classification of mass spectra according to rsp-profile

The generated ascii files were used as input for a previously published custom Python script^20^ with following modification. The low molecular weight rsp L36 (4,452 Da) and L34 (5,378 Da) were often subjected to a peak shift of a few Da, and as a result were missed by our script. We therefore increased the allowed detection mass range for these rsp to 4,445–4,458 Da and 5,370–5,385 Da, respectively.

### Whole-genome sequencing of GBS strains

Whole-genome sequencing (WGS) was performed on 10 randomly picked fish strains (at least 1 per rsp-profile) to confirm rsp-profiles assigned by MALDI-TOF MS analysis. Genomic DNA was extracted with the Wizard Genomic DNA Purification Kit according to the manufacturer’s protocol for Gram-positive bacteria (Promega, USA). Library preparation was done using the Illumina Nextera XT library preparation kit and whole genome sequencing was carried out on an Illumina NextSeq. 500 system at 50× average coverage (Illumina, USA). Genomes were assembled using the metAMOS pipeline (version 1.5rc3).

### *In silico* molecular weight prediction of ribosomal subunit proteins

The 10 generated GBS whole-genome sequences were used for *in silico* extraction of the nucleotide sequences coding for 28 rsp using tBLASTn analysis. The most frequent posttranslational modifications^23^, specifically N-terminal methionine loss and methylation, were considered for subsequent prediction of the monoisotopic molecular rsp weights.

### Average nucleotide identity (ANI) analysis

The phylogenetic relationship between the whole-genome sequenced GBS strains of this study and a collection of publicly available GBS WGS^20^ was assessed by Average Nucleotide Identity (ANI) analysis^24^. ANI was carried out using the Python module PYANI (https://github.com/widdowquinn/pyani), applying the Mummer ANIm method and ANI calculations were performed at sciCORE (http://scicore.unibas.ch/) scientific computing core facility at University of Basel. Euclidian distance matrix calculation and UPGMA hierarchical cluster analysis was performed using the R stats base package. Phylogenetic trees were edited and visualized using the interactive tree of life (iTOL) website^25^.

## Results

### Capsular serotyping

PCR analysis revealed 6 different capsular serotypes in our collection of 249 GBS isolates. The human isolates (171/249) were composed of serotype Ia (N = 35), Ib (N = 22), II (N = 3), III (N = 62), IV (N = 9) and V (N = 12) and 28 non-typeable strains. Fish GBS (63/249) were composed of serotypes Ia (N = 32), III (N = 1), IV (N = 1) V (N = 4) and non-typeable strains (N = 25) whereas, all pig isolates (15/249) were found to be serotype III (N = 15). Ten of the 25 (40%) non-typeable fish GBS and all the pig GBS were tested for multi-locus sequence typing (MLST). All selected non-typeable fish GBS belong to ST7 or its single locus variant (SLV) whereas pig GBS belong to ST651 (N = 8/15), ST862 (N = 1) and 6 strains did not give any sequence type due to assay failure.

### MALDI-TOF MS analyses

#### Identification of 28 pre-defined ribosomal subunit proteins in mass spectra

A total of 249 GBS isolates were measured in quadruplicates, totalling to 996 single spectra. Of the 28 rsp, which we previously found to be reliably measurable by MALDI-TOF MS^26^, 26 could be detected on average per spectrum. Of note, there were few low-quality mass spectra (51 spectra from 41 isolates) with exceptionally small rsp counts (between 7 to 19 rsp). Hence, the median rsp count per measurement was 27 out of the 28 rsp to be detected. Except for L19 (found in 50% of spectra), all rsp were found at high levels across the spectra. L13, L14, L17, L18, L23, L29, L30, L33, L35, S16, S19, S21 and S8 were found in between 92–95% of spectra. L21, L22, L24, L32, L34, L36, L6, S10, S12, S13, S15, S17, S18 and S9 were found in more than 95% of spectra. Visual inspection of some mass spectra with missing L19 indicated that this protein mass was in fact present in the spectra, but its mass peak was rather diffuse and therefore not passing the signal-to-noise threshold set by us.

#### Assignment of GBS isolates to known rsp-profiles

**Figure 1:**
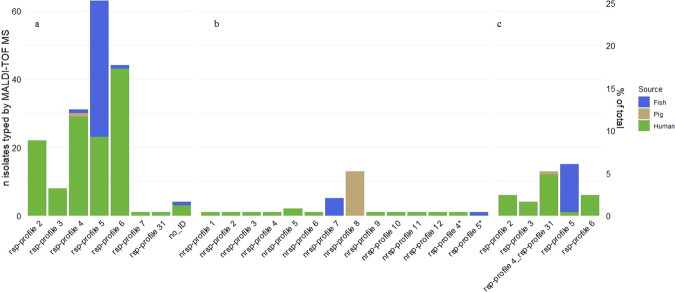
Classification of GBS isolates based on ribosomal subunit protein (rsp) based MALDI-TOF MS analysis. (**a**) Assignment of 170 GBS isolates to rsp-profiles already contained in the reference database. Four isolates were assigned no ID. (**b**) Assignment of 29 GBS isolates to novel rsp (nrsp)-profiles, which were not yet incorporated into our reference database. (*) Two isolates were initially assigned a nrsp-profile but upon visual inspection clearly assignable to rsp-profiles 4 and 5. (**c**) Assignment to known rsp-profiles of 44 GBS isolates for which rsp L19 was not detected in the mass spectrum. Colour coding indicates isolation source of GBS strains (green: human; blue: fish; brown: pig).

A first batch of mass spectra generated from 174 isolates allowed the assessment of how well GBS isolates were assigned to known rsp-profiles by MALDI-TOF MS. Classification according to rsp-profile identity initially failed for 9 isolates. Upon visual inspection of the mass spectra and manual identification of missing rsp, 5 of these 9 isolates could be assigned to an rsp-profile. In total, 170/174 isolates (98%) were successfully assigned to rsp-profiles contained in the reference library. The rsp-profiles 2–6 which stand representative for the globally dominant GBS phylogenetic genotype clusters were also most abundant in our collection (Fig. 1a). Of the 170 classified isolates, 37% (N = 63) were assigned to rsp-profile 5, 26% (N = 44) to rsp-profile 6, 18% (N = 31) to rsp-profile 4, 13% (N = 22) to rsp-profile 2, 4.5% (N = 8) to rsp-profile 3 and single isolate each were assigned to rsp-profile 7 and rsp-profile 31. With regards to the isolation source of the 170 rsp-identified GBS isolates, a clear pattern regarding assigned rsp-profile was seen. While isolates of human origin were found to represent all 7 rsp-profiles covered here, fish isolates almost exclusively fell into rsp-profile 5, with single isolate each displaying rsp-profile 4 and rsp-profile 6, respectively. The sole isolate from pig origin was assigned to rsp-profile 4 (Fig. 1a).

#### Assignment of GBS isolates to novel rsp-profiles

In all four replicate spectra of 31 isolates, one or more rsp were found to display an rsp mass variant not yet contained in our reference database, indicating the occurrence of a novel rsp-profile. Visual inspection of the mass spectra led to the assignment of 2 isolates to the already known rsp-profiles 4 and 5, respectively. The remaining 29 isolates (12% of the 249 isolates) were found to either display previously unknown rsp mass variants (N = 27) or new combinations of known rsp mass variants (N = 2). The novel rsp-profiles contained in these 29 isolates were cross-compared and the distinct profiles termed novel rsp (nrsp)-profile 1 to 12 (Fig. 1b). The most abundant novel profile was nrsp-profile 8 (N = 13), which was exclusively found in GBS strains isolated from pigs which contains ST651. Nrsp-profile 7 (N = 5) of fish GBS and nrsp-profile 5 (N = 2) of human GBS belonged to ST 931 and ST929, respectively **(**Supplementary Table 1**)**. All remaining nrsp-profiles (N = 9) were observed just once in GBS strains isolated from patient samples that belonged to ST24 and ST23, which came out as nrsp-profile 2 and 6, respectively.

#### Classification of GBS isolates with missing L19 mass variant

The overall low measurability of rsp L19 had implications for identification of 44 GBS isolates, in which L19 was missing in all replicate spectra considered for rsp-profile classification. For 13 isolates, the absence of L19 led to the assignment of a double rsp-profile ID (Fig. 1c). Precisely, rsp-profile 4 and rsp-profile 31, which aside from L19 share an identical combination of rsp variants, were identified as closest match, with 27 of 28 rsp detected. For the remaining 31 isolates, the absence of L19 was not an issue, since the detected rsp mass variants allowed unambiguous assignment to a distinct rsp-profile.

#### *In silico* confirmation of rsp-profiles

WGS data from randomly selected 10 fish GBS isolates was used for *in silico* prediction of rsp molecular weights and assignment of MLST identity through the S. *agalactiae* PubMLST website^26^. For all 10 isolates, the rsp-profile assigned by MALDI-TOF MS was supported by the WGS data (Table 1). Eight isolates were assigned to rsp-profile 5 by both MALDI-TOF MS analysis and *in silico* typing. Of these 8 isolates, 1 strain was identified as MLST single-locus variant of ST7 and the remaining 7 isolates as ST7. One isolate was assigned to rsp-profile 4 by MALDI-TOF MS analysis. Due to insufficient sequence quality, only 26/28 rsp masses and no ST could be predicted *in silico* for this isolate. The 26 predicted rsp masses all corresponded to the mass alleles of rsp-profile 4, supporting that this is the true rsp-profile of this GBS strains. One remaining GBS isolate was not assigned to any rsp-profile by MALDI-TOF MS, due to rsp L18 which displayed a mass variant not yet contained in our reference database, supporting the existence of a novel rsp-profile (nrsp-profile 7). This was confirmed by *in silico* analysis, where a previously unknown molecular rsp mass variant of L18 at 12,867 Da corresponds to the peak in the spectra. This novel rsp-profile belongs to ST931, a single-locus variant of the bovine ST591.

**Table 1:** *In silico* confirmation of MALDI-TOF MS assigned ribosomal subunit protein (rsp)-profile of ten Group B *Streptococcus* strains.

Isolate ID	Isolation Source	Serotype	Clonal Cluster (CC)	Sequence type	MS assigned rsp-profile	*In silico* assigned rsp-profiles
CUHK A1	Fish	Ia	7	SLV 7	rsp-profile 5	rsp-profile 5
CUHK A11	Fish	V	7	7	rsp-profile 5	rsp-profile 5
CUHK A12	Fish	Ia	7	7	rsp-profile 5	rsp-profile 5
CUHK A23	Fish	Ia	7	7	rsp-profile 5	rsp-profile 5
CUHK A31	Fish	Ia	7	7	rsp-profile 5	rsp-profile 5
CUHK A41	Fish	Ia	7	7	rsp-profile 5	rsp-profile 5
CUHK A60	Fish	nt	7	7	rsp-profile 5	rsp-profile 5
CUHK A63	Fish	Ia	7	7	rsp-profile 5	rsp-profile 5
CUHK A26	Fish	III	nt	nt	rsp-profile 4	rsp-profile 4*
CUHK A49	Fish	Ia	67	931	nrsp-profile 7	nrsp-profile 7

^*^26/28 rsp predicted corresponded to rsp-profile 4 and 2/28 rsp could not be predicted due to sequence quality, SLV: single-locus variant, nt: non-typeable, nrsp-profile: novel ribosomal subunit protein profile.

#### Correlation between MALDI-TOF MS assigned rsp-profiles and capsular serotype

**Figure 2:**
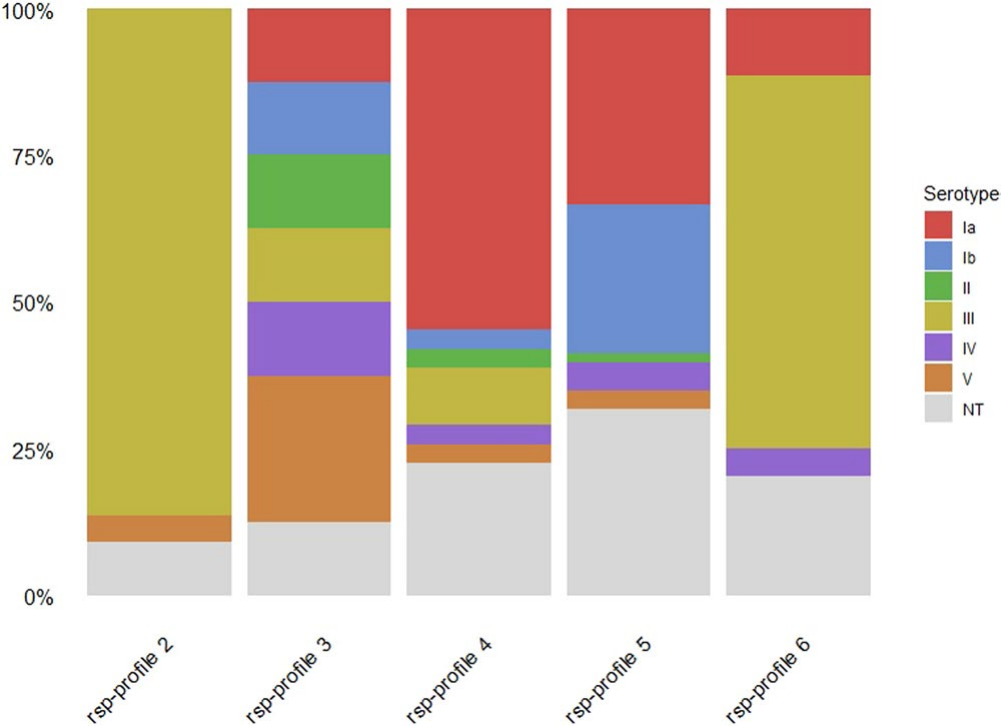
Bar plots visualizing the relation of ribosomal subunit protein profiles 2 to 6 (x-axis) against the associated capsular serotypes of 168 GBS strains.

A total of 168 isolates (human: N = 125; fish: N = 42; pig: N = 1) which were assigned to one of the dominant rsp-profiles 2–6 by MALDI-TOF MS (Fig. 1a) were used to assess the correlation between capsular serotype and assigned rsp-profile (Fig. 2). Strains of rsp-profile 2 and rsp-profile 6 were mostly displaying capsular serotype III (19/22 and 28/44, respectively). Rsp-profile 4 strains were mostly linked to serotypes Ia (17/31) and strains that were rsp-profile 5 either linked to serotypes Ia (21/63) and Ib (16/63) or non-typeable (20/63). Rsp-profile 3 was the most heterogeneous with 1 strain each displayed serotype Ia, Ib, II, III and IV, 2 strains displayed serotype V and 1 strain was non-typeable. The relationship between rsp-profile and serotype observed in our GBS isolates collection correspond to what has been reported in our previous study^20^. The rsp-profiles 2 and 6 are linked to strains with serotype III and rsp-profile 4 is linked to serotype Ia in the global collection whereas, rsp-profile 5, in addition to be strongly linked to serotypes Ia, Ib, shows a stronger link to serotype II globally than in our study. Lastly, rsp-profile 3 was reported to be strongly linked to serotype V and to a lesser degree to serotypes II and IV in the global collection but this could not be supported here due to low number (N = 8) of isolates.

#### Average nucleotide Identity analysis of whole genome sequenced GBS strains

**Figure 3:**
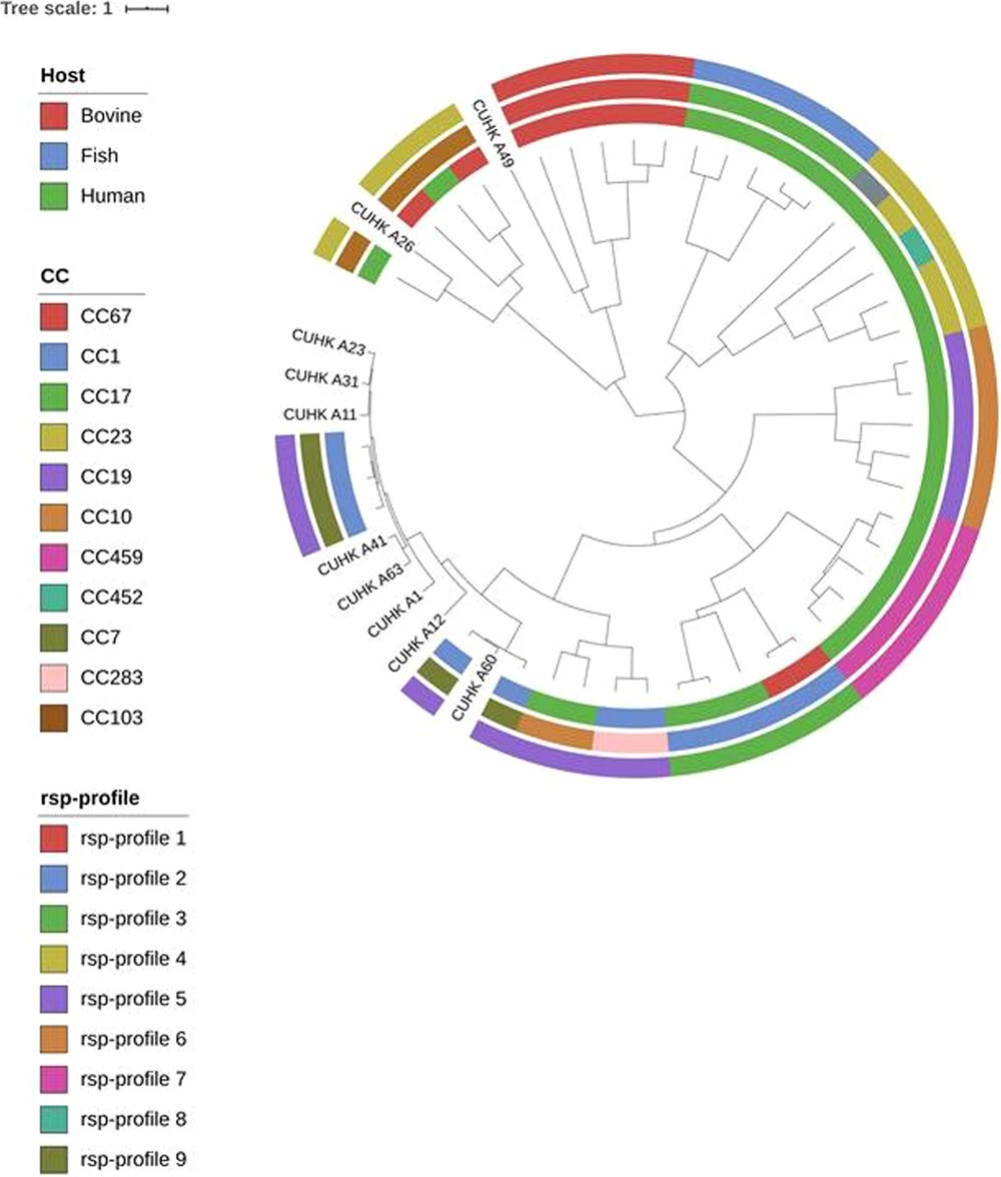
UPGMA phylogenetic tree of 53 GBS strains based on genome-wide average nucleotide identity (ANI) analysis. The set includes ten strains that were whole genome sequenced in this study and 43 reference strains that represent major GBS phylogenetic lineages. Inner circle: Strain host origin Middle circle: clonal clusters Outer circle: rsp-profile identity of reference strains. (Scale bar: nucleotide substitutions per site).

After demonstrating that the rsp-profiles assigned to the 10 fish GBS isolates by MALDI-TOF MS corresponded to the *in silico* typed rsp-profiles (Table 1), average nucleotide identity (ANI) analysis was performed in order to confirm that genotypes displaying identical rsp-profiles share the same phylogenetic background. ANI analysis was carried out using WGS data of the 10 fish isolates and combined with publicly available WGS data of 43 GBS strains that represents the previously reported 6 globally dominant rsp-profiles^20^. ANI analysis grouped the total 53 strains based on genome-wide assessment of inter-strain similarity (Fig. 3). The eight fish isolates (CUHK A1, CUHK A11, CUHK A12, CUHK A23, CUHK A31, CUHK A41, CUHK A60 and CUHK A63) displaying rsp-profile 5 were located within the rsp-profile 5 cluster which harbor GBS genotypes from a broad range of hosts including human and fish species^20^ in the UPGMA phylogenetic tree. The CUHK A26 fish isolate assigned to rsp-profile 4 was grouped together with rsp-profile 4 genotypes of MLST clonal complex (CC) 103 which have been shown to form a genetically highly distinct lineage^20^. The ST931 isolate (CUHK A49), belongs to nrsp-profile 7, and was found to cluster closest to genotypes of the obligate bovine rsp-profile 1 lineage.

## Discussion

The use of MALDI-TOF MS for GBS strain-level typing has previously been demonstrated by the successful identification of hypervirulent GBS genotypes ST17 and ST1 based on detection of genotype-specific protein masses^19,27^. We have recently expanded on these findings and proposed a MALDI-TOF MS method that classifies GBS genotypes based on molecular weight variations of 28 pre-defined rsp. This approach builds on beforehand *in silico* calculated protein molecular masses, moving away from the traditional ‘pattern-recognition’ approach towards targeted, biomarker-based MALDI-TOF MS microbiological identification. The resolution power allows assignment of GBS strains to distinct rsp-profiles, which allows classification of strains according to their core-genome phylogenetic backbone and provides a predictive value regarding probable capsular serotype, virulence capacity or host origin^20^. The application of this method to 249 GBS strains isolated from human and animal sources in Hong Kong aimed to confirm the transferability of this method between different laboratories and MALDI-TOF MS platforms and to investigate the potential of this method for high-throughput screening of novel GBS genotypes, which we hypothesized can be often found in diverse animal host reservoirs. MALDI-TOF MS analyses demonstrate that translation of previously established bacterial sample processing protocol to a different laboratory and different MALDI-TOF MS platform allows for the generation of high-quality mass spectra. All but one of the 28 rsp was reliably measured (27 rsp above 92% and 14 rsp above 95%) in the 996 mass spectra generated here. The overall measurability of the rsp is high, and there were 29 isolates displaying novel rsp mass variants. The only current drawback pertains to the low measurability of the rsp L19, which was found in only half of the spectra. We do not think that this is a general technical limitation of the Microflex platform, given that L19 was still abundant in the other half of the spectra and that by experience a simple repetition of MALDI-TOF MS measurement resulted in the detection of missed L19. Visual inspection of isolates CUHK A23, CUHK A31 and CUHK A60 mass spectra with missing L19 revealed that the main mass variant was present but the peak not distinct enough to pass the signal-to-noise threshold. *In silico* prediction of L19 in these isolates confirmed this observation and can likely be extrapolated to the other isolates with missing L19. L19 is a highly conserved rsp, mostly present as the main mass variant and for the majority of genotypes, missing of L19 does not interfere with the correct assignment of the rsp-profile. The 13 isolates that were assigned with a double-ID due to the missing L19, all matched to both rsp-profile 4 and the rsp-profile 31, which is not a frequently seen profile (1 out of 796 WGS). Furthermore, it has been attributed to a GBS genotype that is located in the same phylogenetic cluster as the majority of rsp-profile 4 genotypes. Hence, the double ID in this case still allows the assignment of genotype to its correct evolutionary relationship^20^.

The classification of the 170 GBS isolates within known rsp-profiles confirmed that our findings of rsp-profiles match with dominant global scale profiles (Fig. 1a) except rsp-profile 1^20^ which was strictly associated with bovine origin. As opposed to PCR serotyping which failed to type 40% of fish isolates, MALDI-TOF MS based typing was very sensitive, with 98% (170/174) of isolates being assigned to a distinct rsp-profile. Comparison of 168 GBS strains from Hong Kong and 523 strains from the NCBI genome database^20^ revealed high concordance between the two collections with regards to association of rsp-profile and capsular serotype (Fig. 2). This further supports the value of the MALDI-TOF MS assigned rsp-profile in providing a predictive measure of likely associated capsular serotypes. In particular, this method will enable (i) rapid identification of hyper virulent CC17 and CC23 strains; (ii) tracking of potential zoonotic GBS since most strains of animal origin display different rsp-profiles than human GBS strains; (iii) first-line screening for specific disease-causing GBS affecting adult populations and (iv) for monitoring the impact of vaccines on the GBS population structure in both human and animals when this becomes available. This method is also rapid into the work flow of routine private or public diagnostic microbiology laboratories that use MALDI-TOF MS equipment for bacterial identification.

A central outcome relates to the 29 (12% of total collection) that were found to display a novel rsp-profile not previously contained in our reference database. Our data supports that rsp-based MALDI-TOF MS can be used to reliably screen such genotypes, which predominantly arise from the under-researched animal hosts. While two of the pig GBS isolates fell into known rsp-profiles (rsp-profile 4 and/or rsp-profile 31), all remaining 13 isolates (87% of all pig isolates) belong to nrsp-profile 8 (Fig. 1b), raising the possibility that these genotypes represent a distinct, pig-associated phylogenetic lineage. Of 63 fish GBS isolates investigated, 86% (n = 54), including eight of the ten WGS isolates (Table 1) fell into the known rsp-profile 5. However, there were 5 fish isolates (8%) that were, upon visual inspection of the mass spectra, found to share a novel rsp-profile 7. WGS based *in silico* confirmation of the rsp-profile in a fish strain (CUHK A49) confirmed the new fish specific lineage nrsp-profile 7 (Table 1). Integration of nrsp-profile 7 into our bioinformatics pipeline followed by re-assignment of the other 4 fish isolates confirmed the presence of nrsp-profile 7 in these strains. This stands representative of how the rsp-based MALDI-TOF MS method can be used for rapid screening of hundreds of isolates, flagging potential novel genotypes which are then subjected to WGS. While only strains with rsp profiles deposited in the reference database can be classified unequivocally, the WGS for strains of these novel genotypes are subsequently used for *in silico* confirmation of the measured 28 rsp followed by the final integration of newly discovered rsp-profiles to the reference database. The ANI phylogenomic analyses conducted here exemplified that the expression of an atypical rsp-profile can be a reliable indicator that a GBS genotype belongs to a very distinct phylogenetic lineage. Two fish isolates (CUHK A26, CUHK A49) which are not belonging to the dominant rsp-profile 5 were found to display a profoundly different phylogenetic background (Fig. 3).

Of less than 400 ppm which is currently below the detection threshold of a routine MALDI-TOF MS machine, and may lead to possible false assignment of rsp-profiles. This may be overcome in future with newer versions of MALDI-TOF MS technology to improving the overall mass range coverage.

## Conclusions

We confirm here the inter-laboratory transferability of a rsp-biomarker based MALDI-TOF MS typing method, its capability to discriminate between GBS genotypes of the major global phylogenetic lineages, its power for rapid screening of hundreds of isolates for surveillance of circulating GBS genotypes and its potential for identifying novel emerging GBS genotypes and atypical clones.

## Supplementary information

Supplementary information.
