# Supplementary material for: A simple, rapid typing method for Streptococcus agalactiae based on ribosomal subunit proteins by MALDI-TOF MS
Source: Sci Rep. 2020 May 29;10:8788. doi: 10.1038/s41598-020-65707-5 (PMC7260235; doi:10.1038/s41598-020-65707-5)
Supplement: Supplementary file 1 — Supplementary information. [file 41598_2020_65707_MOESM1_ESM.docx]

**Supplementary Materials for**

**“A simple, rapid typing method for *Streptococcus agalactiae* based on ribosomal subunit proteins by MALDI-TOF MS”**

Julian Rothen^1,2†^, Dulmini Nanayakkara Sapugahawatte^3†^, Carmen Li^3^, Norman Lo^3^, Guido Vogel^4^, Frédéric Foucault^4^, Valentin Pflüger^4^, Joёl F. Pothier^5^, Jochen Blom^6^, Claudia Daubenberger^1,2*^ and Margaret Ip^3*^

^1^Department of Medical Parasitology and Infection Biology, Swiss Tropical and Public Health Institute, Basel, Basel, Switzerland

^2^University of Basel, Basel, Switzerland

^3^Department of Microbiology, The Chinese University of Hong Kong, Shatin, Hong Kong

^4^ Mabritec AG, Riehen, Switzerland

^5^ Research Group for Environmental Genomics and Systems Biology, Institute of Natural Resource Sciences, Zurich University of Applied Sciences (ZHAW), Wädenswil, Switzerland

^6^ Bioinformatics and Systems Biology, Justus-Liebig-Universität Gießen, Giessen, Germany

^†^ These authors contributed equally to this work.

***Corresponding Authors:** Professor Margaret Ip, [margaretip@cuhk.edu.hk](mailto:margaretip@cuhk.edu.hk) and Professor Claudia Daubenberger, [claudia.daubenberger@swisstph.ch](mailto:claudia.daubenberger@swisstph.ch)

**Supplementary Table 1:** The relationship of the rsp-profiles, serotypes and Sequence types of fish and pig GSB from Hong Kong wet markets

| Host | Serotype (N) | rsp-profile (N) | Sequence type (N) | Remarks |
| --- | --- | --- | --- | --- |
| Fish | Ia (32/63) | rsp-profile 5 (30/32) | ST7 (14/30)  SLV of ST7 (1/30)  ST-Not available (15/30) | 1. 93.7% of serotype Ia fish GBS belong to rsp-profile 5 2. 50% (15/30) serotype Ia, rsp-profile 5 fish GBS belong to ST7 or SLV of ST7 |
|  |  | rsp-profile 6 (1/32) | ST-Not available (1/1) |  |
|  |  | rsp-profile 7 (1/32) | ST931 (1/1) |  |
|  | III (1/63) | rsp-profile 4 (1/1) | ST862 (1/1) | 1. Sample size is too low to make a conclusion |
|  | IV (1/63) | rsp-profile 5 (1/1) | ST7 (1/1) | 1. Sample size is too low to make a conclusion |
|  | V (4/63) | New rsp-profile 7 (4/4) | ST931 (1/4)  ST-Not available (3/4) | 1. 6.3% (4/63) of fish GBS were serotype V and all of them belong to new rsp-profile 7. 2. 25% (1/4) of serotype V, new sp-profile 7 belong to ST931 |
|  | Nontpeables (25/63) | rsp-profile 5 (24/25) | ST7 (3/24)  ST-Not available (21/24) | 1. 39.6% (25/63) of fish GBS were nontypeabe by PCR method. 2. 96% (24/25) of nontypeable fish GBS belong to rsp-profile 5 3. 12.5% (4/24) of nontypeable, rsp-profile 5, fish GBS belong to ST7 4. Need in silico analysis on whole genome |
|  |  | No ID (1/25) | ST-Not available (1/1) |  |
| Pig | III (15/15) | rsp-profile 4 (1/15) | ST-Not available (1/1) | 1. 86.6%% of serotype III fish GBS belong to new rsp profile 8 2. 84.6% of serotype III, new rsp-profile 8 pig GBS belong ST651 |
|  |  | rsp-profile 4_rsp-profile 31 (1/15) | ST651 (1/1) |  |
|  |  | New rsp-profile 8 (13/15) | ST651 (11/13)  ST862 (1/13)  ST-Not available (1/13) |  |
